# Effects of the Community Cats Program on Population Control, Migration and Welfare Status of Free-Roaming Cats in Tokyo, Japan

**DOI:** 10.3390/ani10030461

**Published:** 2020-03-10

**Authors:** Kana Mitsui, Shusuke Sato, Yoshie Kakuma

**Affiliations:** 1 Graduate School of Science & Engineering, Teikyo University of Science, 2-2-1 Senjusakuragi, Adachi-ku, Tokyo 1200045, Japan; 2 Department of Animal Sciences, Teikyo University of Science, 2-2-1 Senjusakuragi, Adachi-ku, Tokyo 1200045, Japan; shusuke.sato.b3@tohoku.ac.jp

**Keywords:** free-roaming cats, community cat program, population, density, feline, behavior, welfare

## Abstract

**Simple Summary:**

The community cats program (CCP), which includes trap–neuter–return activities, has been promoted in Japan to reduce the population of free-roaming cats without harmful effects on their welfare. To ascertain the effects of the CCP, a two-year survey of free-roaming cats was conducted in an area with CCP and another area without CCP in urban Tokyo, Japan. The estimated number of cats was lower in the CCP area than the non-CCP area, but there was no difference in the rate of decline in cat populations between areas. More cats disappeared (moved out, died, or entered homes) than appeared (moved in or born) in both areas in the second year and more males tended to move into the CCP area. There was no difference in the behavior of cats between areas and among seasons. The proportion of cats with poor health was lower in the CCP area than the non-CCP area. These results suggest that the CCP may improve the welfare of free-roaming cats. As the effect of CCP was restrictive in reducing free-roaming cats, the further promotion of neutering of cats may be necessary.

**Abstract:**

The community cats program (CCP), which includes trap–neuter–return activities, has been promoted in Japan to reduce the population of free-roaming cats without harmful effects on their welfare. To ascertain the effects of the CCP, a two-year route census of free-roaming cats was conducted in an area with CCP and the other area without CCP in urban Tokyo, Japan. The estimated number of cats was lower in the CCP area than the non-CCP area, but there was no difference in the rate of decline in cat populations between areas. More cats emigrated or disappeared rather than immigrated in both areas in the second year and more males tended to immigrate into the CCP area. There was no difference in the behavior of cats between areas and among seasons. The proportion of cats with poor health was lower in the CCP area than the non-CCP area. These results suggest that the CCP may improve the welfare of free-roaming cats. As the effect of CCP was restrictive in reducing the population of free-roaming cats, the further promotion of neutering of cats may be necessary to reduce the population density of cats.

## 1. Introduction

Unowned stray cats or free-roaming cats cause hygiene, public health or biodiversity conservation problems by the predation of wild species [1,2,3]. Especially in urban city areas, complaints by local residents about the vocalization or excrement of free-roaming cats concentrated in areas with large numbers of stray cats [4]. Therefore, managing the population of stray cats could lead to human health and welfare benefits, and the conservation of valued species. Free-roaming cats in urban areas are, however, often fed and cared for routinely by compassionate non-owners and this may cause further conflicts between those for protection and those supporting control. 

The number of cats owned in Japan has kept increasing and was estimated at 9,649,000 in 2017, exceeding that of dogs (8,903,000) for the first time [5]. Despite this fact, cats have been euthanized in public animal shelters much more than dogs. According to the “Intake of dogs and cats and accommodation situation of injured animals” published by the Ministry of the Environment, the number of cats that entered municipal animal shelters in 2017 was 62,137 and 82% (50,997) of cats had unknown owners. The total number of dogs and cats euthanized at municipal animal shelters in 2017 was 43,216 nationwide and cats dominated 62% (34,854), and of them, 21,611 were kittens [6]. 

Controlling the free-roaming cat population is also a global challenge. Approximately two to three million cats enter animal shelters per year in the United States, and more than half of the cats are euthanized due to shelter crowding or shelter acquired disease [7]. One of the reasons why many cats were killed was that the majority of these cats were stray or had unknown owners. While the number of cats adopted by new owners is increasing, too many kittens are born to be taken to new homes. Thus, it is considered necessary to reduce the population of stray cats and the number of kittens and to introduce measures to increase the adoption of unowned cats in order to reduce the number of cats euthanized.

Recently, trap–neuter–return (TNR) programs have become popular and have been implemented internationally for the population control of free-roaming cats. The suppressing effects on the cat population by TNR have been reported by simulation studies [7,8] and some other studies have shown the actual decrease of cats by TNR programs [9,10]. In Japan, the Ministry of the Environment, which has the Animal Welfare and Management Office, announced “Guidelines for Proper Keeping of Dogs and Cats in Densed Residential Areas” in 2010 and it recommends that local residents manage cats without owners by neutering and returning or adopting them, aiming to coexist and to control the population of free-roaming cats [11]. The objective of such activities includes eliminating cats without owners in the future. Such cats are called “community cats” and are defined as cats without specific owners, but who are controlled and obtained recognition and agreement by local residents with understanding and cooperation in the area. “Community cats programs (CCPs)” or TNR programs are promoted as an effective method to decrease the number of free-roaming cats. However, there is an argument whether stray cats should be protected or eliminated, and it is necessary to clearly understand their lives, such as their behavior and impact on the environment in order to propose effective management of cats in urban areas [12].

Studies on Japanese stray cats have shown some of the basic features of those cats. Intact males were found to have longer distances of migration than castrated males [13] and home ranges were bigger in males than in females—the biggest was in natural areas, followed in order by rural area, harbors, fishing village and urban cities [14]. It was also shown that the same individuals may repeat immigration and emigration, and it was expected that more males tended to immigrate [15]. Even in the same city, it has been shown that there was a large seasonal variation in the estimates of outdoor cats, and that increase and decrease was repeated, suggesting that the number of cats changed depending on the resources and season in the city area of Osaka, Japan [16]. It was also pointed out that the size of the home range is influenced both by sex and season, and the home range of intact males become larger in the estrus season of females [17].

Seo and Tanida [18] reported that health conditions were judged to be bad in about half of feral cats living in the uptown and downtown areas, and it was presumed that most missing cats died from illness or injury, as opposed to having migrated elsewhere in the west of Japan. Comparing the groups of stray cats with and without a high level of human care in the city area of Tel Aviv, Israel, stray cats receiving a high level of care showed low aggressiveness and low fecal cortisol concentration in spayed females [19]. It turned out that stray cats are influenced by people, and that, even if they were stray, the involvement of people with care leads to the improvement in the welfare of cats.

As mentioned above, activity patterns, ecology and welfare status of stray cats have been studied to some extent, but it has not been thoroughly examined whether CCPs and TNR programs are effective in suppressing the stray cat population, especially in urban areas with problems of feces and urine, and excessive vocalizations of free-roaming cats. Simulation studies have shown that the numbers of adult cats and kittens in the area was reduced by introducing neutering programs; however, there are few studies that show ratios of adult cats and kittens, sexes, behavior, and physical conditions of free-roaming cats based on actual surveys. It is important to determine the ratio of free-roaming cats lost and newly found in an area for effective management of population and nuisance of cats. To contribute to these issues, the aim of this study is to examine the effects of the community cats program on population control, migration, activity patterns and the welfare status of free-roaming cats in an urban city area.

## 2. Materials and Methods

### 2.1. Study Areas

Route censuses of free-roaming cats were conducted for two years in two areas in Adachi City (Ward) in Tokyo Metropolis, Japan. One area was chosen as the CCP area as it was one of two areas registered to Adachi City as the model CCP areas where additional support, such as the higher amount of subsidies for neutering costs, was available from the City Health Care Center. Adachi City, one of 23 specified districts within Tokyo Metropolis, is located north of central Tokyo and the total area of the city is 53.25 km^2^. It consisted of 269 towns (Cho and Chome) and each town had at least one neighborhood association that often-discussed nuisance issues, including controlling free-roaming cats in that area, and became the subjects to implement programs. As a neighborhood association was managing the CCP area, another town within Adachi City, similar to the CCP area, was chosen as the non-CCP area.

Both the CCP and non-CCP areas were located along riverbanks and involved a mixture of stores and houses. According to the data from Adachi City, the non-CCP area was 26.4 ha and the road ratio was 20–25% with no park areas. It had a population of 3802 and 1903 households, whereas the CCP area was 16.0 ha with 15–20% roads and some park areas (less than 5%) and a population of 3225 and 1433 households [20,21]. Approximately two-kilometer census routes were set as there were several streets and corners running through each area where sufficient cats were found in preliminary surveys. Finally, the lengths of the routes were determined as 1.918 km for the non-CCP area and 1.938 km for the CCP area. The route for the non-CCP area starts at 139°47’33” E, 35°45’21” N, while that for the CCP area starts at 139°51’28” E, 35°46’3” N. During preliminary surveys, cat-feeding trays were found at 3 to 4 locations in both areas, but food was more abundant in the CCP area. In the CCP area, registered cats were fed and supplied with water once a day by local resident members voluntarily. Cats were sometimes captured and taken to a veterinary hospital if they were found injured or in poor health, or newly appeared in the area with no sign of being neutered.

### 2.2. Data Collection

The route censuses of free-roaming cats in two study areas were carried out for two years from July 2015 to June 2017. Two surveys were done in a day in an area—one in the morning (from 10 to 12 am) and one in the evening (from 4 to 6 pm). Surveys were carried out on three days for each area in a month, making twelve surveys per month in total.

Since all cats found outdoors were included as free-roaming cats and recorded in this study, they can be either stray, community, or pet cats (owned) that have gone outdoors. Cats with collars were judged as owned and those without collars were assumed to be stray, including community cats.

The observer walked a fixed route for 35 to 40 min in a survey. The data for the CCP area were collected by the first author (K.M.), and that for the non-CCP area were collected by one of the three fixed members (including the first author). When cats were found for the first time outdoors within 5 m on both sides of the streets contained within the route, the location, appearance (coat color and pattern, distinct features), sex if known, adult or kittens by size, ear tipping, collar, health condition (good or poor) and their behavior were recorded according to the description in the “2.3 Data analyses” on survey sheets, and cats were photographed for later identification. A modified version of the survey sheet for stray cat identification developed by Yamane et al. [22] was used. This was adopted based on the assumption that cats could be easily identified by their coat pattern, color, and morphological features, such as tail length and shape. The posture and behavior of the observed cat was described as per its actions when it was found and classified later in analyses.

When the same cats were observed during the census more than once per day, only the time, location and their actions were recorded on the cats’ original sheets. When the photograph could not be taken or was not clear enough to identify the individual, the record of the cat was only used to count “the number of cats found” in later analyses. The sex of the cat was judged by observing its genital area, although tortoiseshell or calico cats were determined as female. Information provided from the local residents was sometimes used to help determine the sex or age of some cats. Ear tipping was used to determine whether each cat was neutered or not. Regarding the health condition of cats, they were judged to be in poor health when they had wounds, scars, nasal or eye discharge, skin inflammation, hair loss, and bleeding even once during surveys.

### 2.3. Data Analyses

Two types of data were used for analyses. The “number of cats found” is the total number of cats recorded for each survey without the identification of individual cats. This number should be regarded as an indicator of an abundance of cats in an area, which may cause a human impression of cat overcrowding. The “number of cats identified per month” is the total number of cats observed and identified, excluding duplicates within the same month based on individual identification records. Using the total number of cats identified per month, linear regressions were performed in two areas and were compared by a covariance analysis. The cumulative number of cats identified was calculated as the total number of cats identified throughout the study period, excluding duplicates. The individual cats, which moved in and out of each area in the second year, were also identified and counted.

The estimated population was calculated by the capture–mark–recapture method using the number of cats identified per month in each area using data from the first year. This method can estimate the number of individuals by capturing animals, attaching marks, releasing them, and capturing them again, and it can be applied to various animal species [23]. Here, applying the Lincoln–Petersen method, *N* is the estimated total population, *m*^1^ is the initial number of cats identified (the total number of marked individuals in that month), *n*^1^ is the number of cats found in the next month, and *m^2^* is the number of cats identified in the next month (the number of marked individuals found in that month).

(1) $$\frac{m^{1}}{\;N}=\frac{m^{2}}{n^{1}}$$

(2) $$N=n{\;}^{1}\times \frac{m^{1}}{m^{2}}$$

The population density (the cumulative number of cats identified/area), neutering rate (the number of cats with ear tipping/cumulative number of cats identified), and poor health rate (the number of cats in poor health/cumulative number of cats identified) were calculated for each area in the same way as in previous studies [18,22].

The behaviors of cats were analyzed using records obtained from surveys of the second year, from July 2016 to June 2017. Descriptions of the behaviors of cats at their first encounters were classified into the following 11 items according to previous studies [24,25]: resting, feeding, grooming, exploring, excreting, scratching, urine spraying, rubbing, nursing, playing, and pursuing other cats. When the same cats were observed more than once a day, and when their behaviors were different, they were counted separately. The year was divided into four seasons, each containing three months (e.g., spring is from March to May), and the behaviors of cats were compared between two areas and among four seasons using chi-square tests.

Statistical analyses, in order to compare the two areas, were done with paired t-tests, chi-square tests, or Fisher’s exact test. BellCurve for Excel 3.20 (Social Survey Research Information Co., Ltd., Tokyo, Japan) was used for statistical analyses.

## 3. Results

### 3.1. Numbers of Cats in Each Area

#### 3.1.1. Observed and Estimated Numbers of Cats

The total number of cats found in each area throughout the survey period was 981, including 116 kittens in the non-CCP area, while 593, including 7 kittens, were found in the CCP area. The sum total of cats identified per month for the survey period was 686 (including 84 kittens) in the non-CCP area, whereas 355 (including three kittens) were identified in the CCP area.

The cumulative number of cats identified was 160, including 42 kittens for the non-CCP area, and 59, including two kittens, in the CCP area. The cats that could not be identified by defects, such as those without photographs, were excluded from the results.

The estimated number of cats per month was calculated for each area in the first year. It ranged from 112 to 188 for the non-CCP area, and from 43 to 56 for the CCP area. The number of cats in each area was considered to be within these ranges, while the number of cats in the non-CCP area was estimated to be two to three times as many as in the CCP area. The cumulative number of cats identified for the first year was 137 for the non-CCP area, and 46 for the CCP area, and these were in fact within the range of the estimates for both areas.

Using the cumulative number of cats identified over two years for calculation, the population density of the cats was higher in the non-CCP area (6.1 cats/ha) than in the CCP area (3.7 cats/ha).

#### 3.1.2. Comparison of Monthly Variation

**Figure 1:**
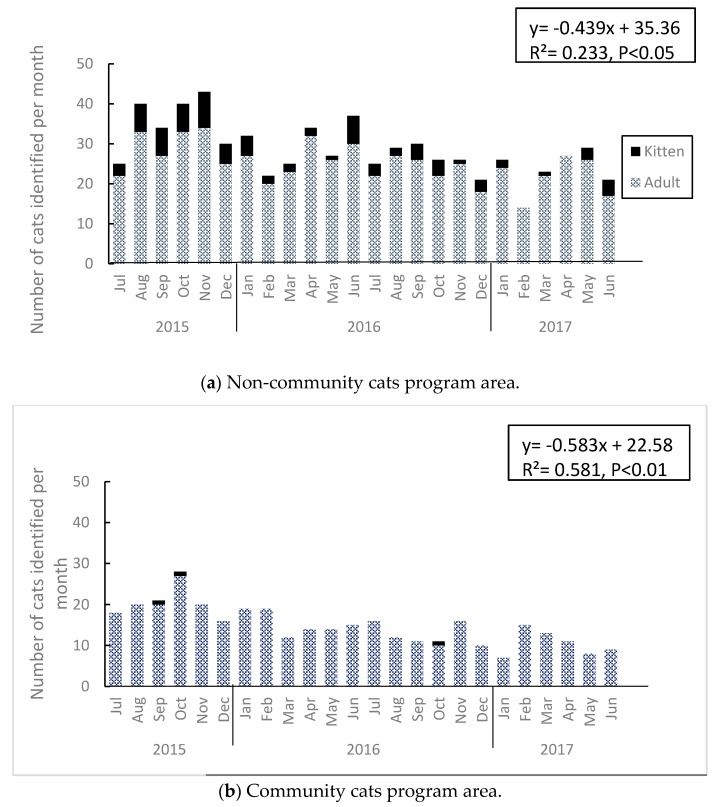
Variations in the number of free-roaming cats identified per month in the community cats program (CCP) area and non-CCP area from July 2015 to July 2017: (**a**) no-CCP area; (**b**) CCP area.

Figure 1 shows the monthly variation of the total number of adult cats and kittens identified in both areas for two years. The number of cats in the non-CCP area was the highest in November 2015, and the lowest in February 2017, while the number of cats in the CCP area was the highest in October 2015, and the lowest in January 2017. While these peaks and bottoms were slightly mismatched, the number of cats observed reached the maximum in autumn and the minimum in winter.

Kittens were seen every month in the non-CCP area, except for February and April 2017, while in the CCP area, they were only found in three months: September and October 2015 and October 2016.

#### 3.1.3. Comparison between Areas

When linear regressions were performed using the total number of identified cats per month for each area, the following formulae were derived: y = −0.439x + 35.36 (R² = 0.233, *p* < 0.05) for the non-CCP area; and y = −0.583x + 22.58 (R² = 0.581, *p* < 0.01) for the CCP area (Figure 1). Regression coefficients were negative for both areas, showing a decline in the number of cats in both areas. While the slope was slightly larger for the CCP area and the decrease rate was higher, the test to show the difference was not significant (covariance analysis, *p* > 0.05). Both areas showed a decrease of 5–6 cats per year.

As for the number of adult cats identified per month, there were significantly more cats in the non-CCP area, as compared to the CCP area (paired *t*-test, *t* = 9.89, df = 23, *p* < 0.01). The average ± standard deviation (SD) for the non-CCP area was 25.0 ± 5.1, and 14.6 ± 4.7 for the CCP area. As for the number of kittens identified per month, there were significantly more kittens in the non-CCP area, as compared to the CCP area (paired *t*-test, *t* = 7.01, df = 23, *p* < 0.01). The average ± SD for the non-CCP area was 3.5 ± 2.4 kittens, and 0.1 ± 0.3 for the CCP area. The total number of cats was significantly larger in the non-CCP area than in the CCP area (paired *t*-test, *t* = 11.5, df = 23, *p* < 0.01).

### 3.2. Migration of Cats

**Figure 2:**
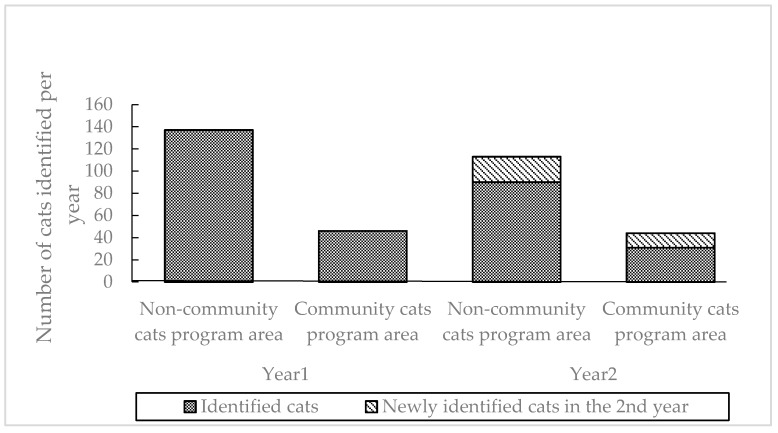
Yearly changes in the total number of cats identified in areas with and without community cats programs. Dark gray columns show the cats observed in Year 1 and light gray parts show newly observed cats in Year 2.

Yearly changes in the total number of cats identified in areas with and without CCP are shown in Figure 2. More cats disappeared than immigrated. The number of immigrants was 23 in the non-CCP area, and 13 in the CCP area, whereas 46 cats disappeared from the non-CCP area and 15 disappeared from the CCP area. Thirty-three percent of cats in the CCP area and 34% of cats in the non-CCP area were not found in the second year, but 28% of cats in the CCP area and 17% of cats in the non-CCP area were newly identified; thus, a higher proportion of cats were new in the CCP area.

When the number of adult cats and kittens that migrated and stayed was compared, the main factor determining the appearance and disappearance seemed whether the cat was an adult or not; however, there was no significant difference between areas. The number of male, female, and neutered cats that appeared and disappeared in both areas was compared, and there was a significant difference between immigration and stay rates in males (*p* = 0.008); nevertheless, more males immigrated into the CCP area (Table 1).

### 3.3. Proportions of Cats Ear-Tipped, with Poor Health, or Collared

Of the identified cats, 15% (*n =* 24) were ear-tipped, 84% (*n =* 136) were not ear-tipped, and 1% (*n =* 2) was unknown in the non-CCP area. In the CCP area, 44% (*n =* 26) were ear-tipped, 51% (*n =* 30) were not ear-tipped, and 5% (*n =* 3) were unknown. The proportion of ear-tipped cats, that is, the neutering rate, was much higher in the CCP area than in the non-CCP areas, and a significant difference was found by a chi-square test (df = 1, χ^2^ = 23.0, *p* < 0.01).

Twenty-eight percent of cats were in poor health (*n =* 45), whereas 72% were in good health (*n =* 115) in the non-CCP area. Meanwhile, only 7% were in poor health (*n =* 4) and 93% were in good health (*n =* 55) in the CCP area. The number of cats in poor health was significantly higher in the non-CCP area than in the CCP area (df = 1, χ^2^ = 11.3, *p* < 0.01).

In the non-CCP area, 9% (*n =* 14) were wearing collars and 91% (*n =* 146) were not. In the CCP area, 7% (*n =* 4) were wearing collars and 93% (*n =* 55) were not. The number of cats with collars was larger in the non-CCP area than in the CCP area, among the identified individuals, but no significant difference was seen in the ratio (df = 1, χ^2^ = 0.22, *p* = 0.6).

### 3.4. Behavior of Cats

Nine behavioral categories, except for “urine spraying” and “rubbing”, were observed in the eleven items in both areas. Seventy-five percent (*n =* 267) of cats were “resting”, 12% (*n =* 91) were “exploring”, 7% (*n =* 29) were “grooming”, and 2% were “feeding” in the non-CCP area. In the CCP area, 77% (*n =* 174) were “resting”, 14% (*n =* 54) were “exploring”, 6% (*n =* 15) were “grooming”, and 3% were “feeding.” Four categories of “resting”, “feeding”, “grooming” and “exploring” were compared between the areas and seasons by chi-square tests, and there were no significant differences (*p* > 0.05). Seven other behavioral categories, such as “excreting”, “scratching”, “urine spraying”, “rubbing”, “nursing”, “playing”, and “pursuit of other cats” were rarely observed and were excluded from the statistical analyses.

## 4. Discussion

It was revealed that the number of free-roaming cats, both adult and kittens, was smaller in the area with a community cats program, as compared to the area without such a program in urban city residential areas in Tokyo. It has been suggested that TNR programs have suppressive effects on the number of stray cats in some previous studies [9,10]. The decrease rate of cats in the CCP area was, however, found not to be different from that in the non-CCP area, meaning that the suppressive effects of CCPs were restrictive. The fact that a high proportion of adult cats, rather than newly born kittens, affected the number of cats was consistent with the findings from a previous study that reported a low survival rate (43% in 7 months) of stray cats’ kittens in Japan [26].

The population densities of cats, calculated from the number of identified cats throughout the study period, were less than those in New York City (urban residential area), United States [24]. The population density in the non-CCP area (6.1 cats/ha) was close, but slightly less than, those of residential and shopping areas (6.5–6.8 cats/ha) in Kitakyushu City, Fukuoka Prefecture [22], and the density in the CCP area (3.7 cats/ha) was less than that in a residential area in Onomichi City (5.8 cats/ha), Hiroshima Prefecture [18], Japan. These findings suggest that the lower density of cats in the CCP area in urban residential area in Tokyo could be due to the CCP.

The number of cats in both areas declined because more cats disappeared than immigrated. It was also revealed that about 34% of cats found in the non-CCP area, and about 33% in the CCP area, disappeared by the second year. It was reported that there was no significant difference in the number of lost cats between the two areas when CCP was not implemented [18], suggesting that CCP did not influence emigration. While these emigrant cats included cats that migrated, died, and those that were picked up by humans, migration may be considered to be more frequent, according to a previous study, which reported 6% mortality over 11 years in community cats in the US [9]. The CCP on the streets, however, may be more vulnerable to traffic accidents, fighting and infectious diseases due to intruder cats and other animals. Our long-term survey of free-roaming cats, identified in the same area, and detailed analyses of the migration of cats, highlighted the importance of controlling the migration of cats in the CCP. There was a significant difference between the areas, with respect to males, when new individuals joined the area, and new males were hard to find in the non-CCP area; however, the home ranges of cats have been reported to be broader in males than in females [14,27]. This may suggest that the existence of more healthy males prohibits the intrusion of males from outside areas. Otherwise, CCPs may cause a “vacuum” effect, as pointed out for TNR [28,29], which means that new cats could be drawn to the area where resident cats were neutered or removed.

While the neutering rate in the CCP area, that is, the proportion of cats with ear-tipping, was 44%, the number of cats was suppressed. It has been estimated that a neutering rate of 51–94% is required to reduce the population [7], but our results suggest that the number of cats may decrease even if the neutering rate is not very high. In the CCP area, kittens were hardly observed, suggesting that the birth of kittens was clearly suppressed. It was also assumed that the reason for less observational data being acquired for kittens in the CCP area could be due to the kittens being lost because of adoption by human volunteers who were taking care of the cats, especially since the neutering rates, estimated to be the rates of cats with ear-tipped, suggest that kittens may be born in both areas.

This study provides a valuable report examining the effects of CCP on the welfare status of cats in regards to their health conditions and the behavior of free-roaming cats. In the CCP area, the proportion of cats in poor health was much less, as compared to the non-CCP area. In a study in areas without CCPs in Onomichi, Hiroshima, about half of the free-roaming cats were considered to be in poor health [18]. There could be great regional differences in the welfare status of stray cats, and cats in urban Tokyo may be better cared for, even without CCPs, but the CCP was shown to improve the welfare of free-roaming cats considerably.

There was no difference in the rate of collar wearing between the areas. This may be due to the fact that the number of cats wearing collars was small, even in the CCP area, and more people who owned cats kept them indoors in urban areas. Our finding is consistent with the recently reported survey results from the Tokyo Metropolitan government, which estimated the rate of outdoor cats wearing collars to be 9%, and reported that it has continued to decrease, whereas the rate of indoor-only owned cats was as high as 74% [30]. The number of cat intakes was reported to be smaller in the area where TNR was implemented than in the area without TNR; thus, TNR has been shown to raise human residents’ awareness of free-roaming cats [10].

In this study, the behaviors of stray cats in an urban area were directly observed, and overall, were revealed to be generally quiet. There were no significant differences between the areas and among seasons in the behavior of cats, despite the expected differences in food availability, reproductive status and direct interactions with humans. While human activity was shown to affect the amount and daily rhythm of cat activity [31], it cannot be applied to free-roaming cats, probably because the relationship between local people and unowned cats is weak. Since nearly 80% of cats were resting when observed, it may be more suitable to carry out the behavioral observation of focal cats for a longer time, as other studies have done [32], to see the effects of CCP in detail and in regards to the behavior of free-roaming cats.

## 5. Conclusions

In conclusion, this study showed the suppression of populations, population densities and the estimates of free-roaming cats in the area with the CCP. The CCP was also shown to have a positive effect on the welfare of cats by increasing neutering rates and improving health conditions; however, it did not change the behaviors of cats. To examine the effects of the CCP further, it is also necessary to investigate how the CCP affects cats, from both a human and cat perspective. The CCP may increase local residents’ awareness of animal welfare.

While the CCP is promoted in Japan, and other countries, as a major strategy to control free-roaming stray cats, higher neutering rates may be necessary to steadily reduce the population. Since several feeding stations were found at places other than designated areas for the registered CCP area during the survey, further management of resources, including feeding by humans, will be important in order to suppress the population of free-roaming cats effectively.

## Figures and Tables

**Table 1:** Number of cats that were lost, stayed, and immigrated from Year 1 to Year 2 in the community cats program (CCP) and the non-CCP areas.

Category of Cats		Number of Cats		Cramer’s V	*p*-Value (Fisher’s Exact Test)
		Non-CCP Area	CCP Area		
Adults	Lost	33	14	0.003	1.000
	Stay	72	31		
	Immigration	14	12	0.137	0.162
Kittens	Lost	13	1	0.206	0.424
	Stay	19	0		
	Immigration	9	1	0.260	0.345
Male	Lost	10	1	0.128	0.433
	Stay	33	1		
	Immigration	3	3	0.560	0.008 *
Female	Lost	18	5	0.012	1.000
	Stay	44	13		
	Immigration	6	1	0.064	1.000
Neutered	Lost	2	2	0.034	1.000
	Stay	13	16		
	Immigration	0	1	0.162	1.000

The number of cats that were lost and stayed, and those that immigrated and stayed was, respectively, compared between the CCP and the non-CCP areas. * There was a significant difference between immigration and stay rates in males.
